# Alexander Laatsch: Biochemisches Rechnen: Ein Übungsbuch für das Medizinstudium

**DOI:** 10.3205/zma000943

**Published:** 2015-02-11

**Authors:** Johannes Schulze

**Affiliations:** 1 Goethe-Universität Frankfurt/Main, Institut für Arbeits-, Sozial- und Umweltmedizin, Frankfurt/Main, Germany

## Recension

This thin volume of 56 pages is the book I wish I had written myself since my experience corresponds closely with that of the author. Medical students generally have difficulty dealing with formulas and intermediate level mathematics often cause problems. Calculations receive only a limited amount of attention during medical studies, but they are used consistently during the preclincal study..

This book offers structured exercises beginning with calculating dimensions (chapter 1), continues with seemingly complex, but simple calculations (chapters 2 – 4: exponentiation, percentage calculations and rule of three) and applies these calculations to examples from chemistry and biochemistry. It starts out very simply and acknowledges that calculations must be explained in detail, something borne out by my own experience. Using this book, students are spared embarrasment asking about basic math steps. Example questions specific to the material covered are at the end of each chapter; detailed answer keys make it easier to understand not only the correct approach, but also the solution. Based on my seminar classes and role as a reviewer for publications, I can confirm that elementary calculations must be repeated and practiced.

This book gives students the opportunity to bring themselves up to an acceptable math level on their own. The level achieved by the end of the book should suffice for comprehending the basics of chemical and biochemical calculations; it is, however, certainly not sufficient for understanding physical calculations. The book is conceived as a workbook and I would appreciate to see a greater number of detailed exercises. Although (additional) examples are presented in practicals and seminars, the individual solution, particularly the approach to solving a problem, is often presented (too) quickly. To understand methods of calculation, it also seems necessary to differentiate more clearly between alternatives solutions when explaining a solution. In addition, aspects of accuracy which result from using decimal places (exercise 3.4) are more likely to cause confusion in novices and should be covered separately.

In some places it is noticeable that the book was not written by a physician. In the case of suspected pancreatitis, determination of the lipase activity is relevant; in terms of complications regarding cholestasis, the alkaline phosphatase is important. The complexity of question 9.2 remains unchanged if the phrasing of the problem would take this into account. More relevant are inaccuracies in clinical examples and that are referred to in this book. For instance, the clinical description of 75% stenosis of an artery refers to the area, not the diameter. In exercise 3.4, the diameter is given and the change seen as percent of the diameter, which does not correspond with the percentual change in area.

I hope this book not only finds a widespread acceptance among students who have difficulty with biochemical (or medical) calculations, but also will be expanded to include chemical calculations and the transformation of a math problem into a formula. Perhaps then I will not have to repeat the basics of physical and chemical dimensions in the first semester of the clinical studies.

## Competing interests

The author declares that he has no competing interests.

